# Amino Acid Similarity Accounts for T Cell Cross-Reactivity and for “Holes” in the T Cell Repertoire

**DOI:** 10.1371/journal.pone.0001831

**Published:** 2008-03-19

**Authors:** Sune Frankild, Rob J. de Boer, Ole Lund, Morten Nielsen, Can Kesmir

**Affiliations:** 1 Center for Biological Sequence Analysis, Technical University of Denmark, Lyngby, Denmark; 2 Theoretical Biology/Bioinformatics, University of Utrecht, Utrecht, The Netherlands; 3 Academic Biomedical Centre, University of Utrecht, Utrecht, The Netherlands; AIDS Research Center, Chinese Academy of Medical Sciences and Peking Union Medical College, China

## Abstract

**Background:**

Cytotoxic T cell (CTL) cross-reactivity is believed to play a pivotal role in generating immune responses but the extent and mechanisms of CTL cross-reactivity remain largely unknown. Several studies suggest that CTL clones can recognize highly diverse peptides, some sharing no obvious sequence identity. The emerging realization in the field is that T cell receptors (TcR) recognize multiple **distinct** ligands.

**Principal Findings:**

First, we analyzed peptide scans of the HIV epitope SLFNTVATL (SFL9) and found that TCR specificity is position dependent and that biochemically similar amino acid substitutions do not drastically affect recognition. Inspired by this, we developed a general model of TCR peptide recognition using amino acid similarity matrices and found that such a model was able to predict the cross-reactivity of a diverse set of CTL epitopes. With this model, we were able to demonstrate that seemingly distinct T cell epitopes, i.e., ones with low sequence identity, are in fact more biochemically similar than expected. Additionally, an analysis of HIV immunogenicity data with our model showed that CTLs have the tendency to respond mostly to peptides that do not resemble self-antigens.

**Conclusions:**

T cell cross-reactivity can thus, to an extent greater than earlier appreciated, be explained by amino acid similarity. The results presented in this paper will help resolving some of the long-lasting discussions in the field of T cell cross-reactivity.

## Introduction

Each T cell expresses thousands of T cell receptors (TCR) of a single specificity that allows inspection of peptide fragments bound by major histocompatibility complex molecules (MHC) on the surface of other cells. Peptides originate as the product of intracellular protein turnover, and both foreign and self-peptides are able to form peptide:MHC complexes (pMHC). Presentation of peptides for which the inspecting CTLs have not been tolerized, triggers a cytotoxic response. Although much has been learned about peptide processing and MHC presentation [1], [2] it is still largely unknown why roughly half of all natural foreign pMHC are ignored [3], [4]. The processing and MHC binding of naturally processed foreign peptides is a primary requirement for the initiation of a cellular immune response. However, the availability of a suitable TCR further determines if a peptide is immunogenic. The structural mechanism of T cell recognition is a highly debated subject in the immunological literature and a consensus view of the promiscuous peptide recognition has not yet been reached (see e.g., [5]). The core problem is that T cells seem to combine high specificity with the ability to recognize a surprisingly large number of dissimilar antigens. Two terms are often used to describe this nature of T cell recognition. **Poly-specificity** is used to emphasize TCR's ability to recognize multiple distinct/unrelated pMHC ligands with high specificity (with little or no tolerance to substitutions of the ligands) [6], [7]. **Cross-reactivity** is a term that was originally used to indicate unexpected reactivity to targets that differed from those used to initially define the T cell clone [8]. Several studies suggest that T cells can recognize seemingly dissimilar epitopes (for a summary see [6]), while other studies have established that substitutions affect peptide recognition in a predictable and additive manner [9] suggesting that the majority of cross-reactive pMHC complexes share structural similarities. One outstanding question in T cell biology is therefore whether T cell cross-reactivity is mostly a stochastic phenomenon induced by unpredictable structural constraints or, whether we can predict which peptides should be cross-reactive. Previous studies of cross-reactivity have focused on limited data covering a single or a few T cell clones. Here, we investigate a simple model of T cell cross-reactivity and perform a large-scale analysis spanning both a broad set of experimental settings, heterogeneous pathogens, MHC molecules and T cell clones. We use this benchmark to investigate whether cross-reactivity is either generally predictable or mostly random. Finally, we test whether the degree of host mimicry is negatively correlated with immunogenicity. By analyzing a large set of known HLA-A2 restricted HIV epitopes, we investigate if potential HIV epitopes with high similarity to self are able to trigger detectable immune responses. Our results suggest that amino acid similarity, rather than identity, is a predictive measure of cross-reactivity.

## Results

### Visualizing TCR recognition sensitivity toward single mutations

**Figure 1:**
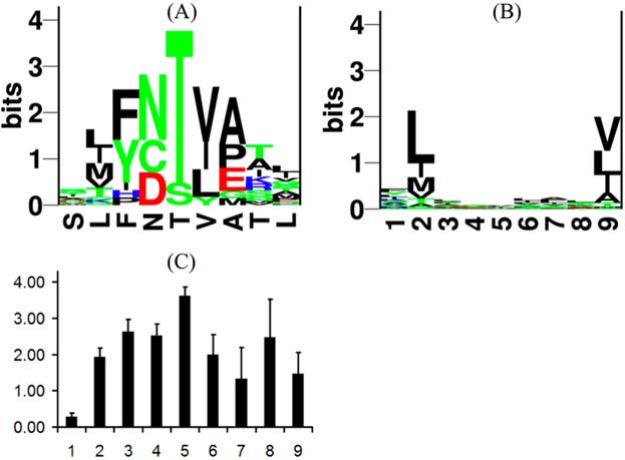
TCR and MHC binding motifs. (A) Logo plot [11] of recognized single variant peptides for CTL clone T4. The x-axis shows the positions in SLFNTVATL. The letters stacked on top of each position are the accepted amino acid substitutions. The y-axis gives Shannon information [14] which is a measure of how conserved a position is. Rigid positions have few but tall letters, while very degenerate positions have many but very short letters. For example, position 1 was mutated 19 times of which 18 variants preserved TCR binding, only the S1R variant compromised TCR binding while the MHC binding was preserved (see [38]). The frequency of amino acids occurring in this TCR motif can also be used to estimate the number of distinct ligands this T cell clone can recognize (see text for details). (B) Sequence motif of HLA-A2 binding peptides (277 HLA-A2 restricted peptides were extracted from the SYFPEITHI database [15]). (C) The average Shannon information at each position, for the CTL clones: G10 and T4, and PBMC.

We analyzed public data on CTL sensitivity and created a visualization of how CTLs react to single amino acid substitutions. Lee et al. [10] analyzed the specificity of CTL responses against the immunodominant HLA-A2 restricted HIV Gag epitope SLFNTVATL (SFL9). IFNγ production was measured in response against all 171 single mutant variants of SFL9. Abrogated TCR responses were mostly due to loss of TCR binding as the majority of SFL9 variants retained binding to MHC. The cross reactivity data for the three data sets: G10, T4 and PBMC were converted into a position-specific-scoring-matrix (PSSM) as described in Materials and Methods. The recognition motif of the T4 clone (the PSSM matrix) is visualized in Fig. 1A as a Logo plot [11]. The plot shows a stack of the possible amino acid mutations on each position in SLFNTVATL (x-axis). The height of the stack is reciprocal to the number of tolerated mutations (i.e., it indicates the degree of T cell recognition specificity at this position, see Materials and Methods). Few tolerated mutations translate into tall stacks while many tolerated mutations show up as short stacks (bars). For example, in position five, only one variant is tolerated (T5S) and is shown as a tall bar. The remaining variants on position 5 were unable to bind the TCR even though the binding to MHC was mostly preserved. On the contrary, in position one, 18 out of 19 variants preserved the TCR recognition. The logo plot for CTL clone T4 given in Fig. 1A suggests that the central peptide positions are most important for peptide-TCR binding, which is in agreement with earlier data [12], [13]. The average Shannon information [14] plot for T4, G10 clones and PBMC is shown in Fig. 1C. This figure also indicates that positions 2–6 and 8–9 are most important for peptide recognition whereas position one is consistently of little importance. Position 2 and 9 are the main positions determining peptide binding to the HLA-A*0201 molecule, see Fig. 1B. Thus, positions 3–6 and 8 were consistently involved in the primary TCR recognition motif. Moreover, the sequence motif for T4 clone suggests that tolerated substitutions tend to be conservative with respect to the original epitope sequence, SLFNTVATL. Examples are F3Y (both non-polar and aromatic), T5S (both polar), and V6I (both aliphatic). Similar observations on tolerated substitutions were made for the other two CTL clones (data not shown). Taken together, these data suggest that amino acid similarity could be a major component of T cell recognition.

### How many different ligands can a TCR recognize?

Using the information in the TCR amino acid position specific scoring matrices, we estimate the number of ligands recognized by a given T cell clone by assuming that recognizable peptides contain only those amino acids giving a detectable ELISPOT response in the Lee et al. [10] study. Non-recognized peptides are the ones containing at least one prohibited amino acid for which no response was detected. The number of recognizable peptides was computed by the following procedure. The degeneracy of a TCR on a single position was measured as the diversity of amino acids present at that position defined in terms of the Simpson index (see Materials and Methods). This diversity measure yields a value between 1 and 20. Here, 20 means that all amino acids are used with equal frequency at a position, and 1 means that only a single amino acid is found. The higher the diversity the more degenerate the TCR is at this position. In the binding motif of T4 clone (Fig. 1A) the first position diversity is very high, 13.26, as expected, because this position is highly degenerate. In the conserved position five, the Simpson diversity drops to 1.29. The product of the tolerated amino acid diversity at each position can provide an estimate of the number of ligands a T cell clone can recognize. For T4, we estimate a total of 5.6⋅10^5^ ligands in this way and this value is in good agreement with previous estimates [8]. For the G10 clone, we estimate 3.2⋅10^6^ ligands, suggesting that this clone is more degenerate. Similarly, one can estimate the number of ligands that can bind to a MHC molecule. For example, the HLA-A*0201 molecule (see Fig. 1B for the binding motif) can bind 4.8⋅10^9^ distinct peptides [15]. Thus, measured in this way the CTL binding event is three orders of magnitudes more specific than that of the MHC.

### CTL cross-reactivity modeled by peptide similarity

The above calculation suggests that a single T cell receptor can recognize as many as 10^6^ ligands. How related are these ligands, and is the cross-reactivity of a T cell clone predictable? A few studies suggest that cross-reactivity is not completely random [9], [16], while others argue that T cells can recognize unrelated ligands (see e.g. [17]). Here, we investigate whether TCR peptide cross-recognition can be predicted by a quantitative model of peptide similarity using amino acid similarity matrices (SM) as explained in detail in Materials and Methods. The peptide similarity score is unity for two identical peptides, and 0 for peptides of maximum dissimilarity, as defined by the SM. Note, that this simple model does not differentiate between positions. Below, the predictions made from this model are tested on several independent data sets, and compared against the performance of random predictors.

### Predicted cross-reactivity of SLFNTVATL variants

**Figure 2:**
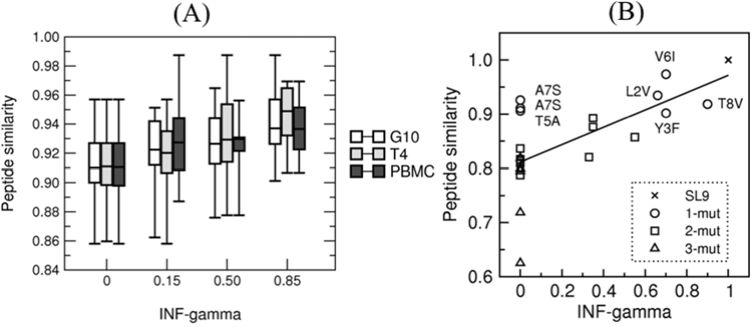
Predicting cross-reactivity. (A) Box plot of ELISPOT data for the two CTL clones G10 and T4, and PBMC. The x-axis shows the relative IFNγ secretion measured for 171 single mutants of SLFNTVATL (SFL9). Immunogenicity was grouped in four bins with average ELISPOT responses of 0, 0.15, 0.50 and 0.85 of maximal ELISPOT for SFL9. In both figures the y-axis shows the predicted CTL recognition in terms of BLOSUM35 similarity scores (see Eq. 2). Unfavorable (non-conservative) substitutions (low x) are associated with a low similarity score (low y) whereas conservative substitutions (high x) in general are associated with higher similarity scores (high y). (B) Observed and predicted recognition of patient derived SLYNTVATL (SYL9) variants with 0–3 mutations. The axis shows the relative IFNγ and peptide similarity scores. Note, that the IFNγ response falls to a half when peptide similarity is around 0.85.

Fig. 2 shows box plots of the level of IFNγ response of three CTL clones in response to stimulation with the 171 variant peptides of SLFNTVATL (data from Lee et al. [10]). The Pearson correlation coefficients between their relative SFU and our peptide similarity score were: 0.40, 0.39, and 0.35 for G10, T4, and PBMC data, respectively (p<0.0001, Monte Carlo randomization exact estimate). Since PBMC consist of two clones, where one clone is dominant [10], the prediction performance on this data set is similar to the performance on the single clonal data. These significant correlation coefficients suggest that peptide cross-reactivity can, to some degree, be estimated from peptide similarities. Thus the proposed model of peptide similarity is capable of producing significant predictions of the loss of recognition due to single amino acid substitutions. Iversen et al. [18] measured IFNγ secretion by T cells specific for SLYNTVATL (SYL9), when they are stimulated with naturally occurring (i.e, patient derived) variants of SYL9. Data consisted of 21 variants of SYL9. Each variant peptide had between 1 and 3 mutations with respect to SYL9. Fig. 2B presents a scatter plot of the data from Iversen et al. [18] for the T4 clone, where the peptide similarity is plotted on the y-axis against the relative IFNγ secretion (x-axis). Using the BLOSUM35 matrix to calculate the peptide similarity score (see Materials and Methods) the Pearson correlation was 0.65. Similar results were obtained using BLOSUM matrices 35–90 (data not shown). For the remaining CTL clones (G10, C-3, C-4, C-22 and C-32) tested by Iversen et al. [18] correlations were 0.49, 0.47, 0.55, 0.60 and 0.57 respectively (all values are significantly different from zero with p<0.02 Monte Carlo randomization exact estimate). This model of peptide similarity (or cross-reactivity) was thus able to explain around 20−40% of the IFNγ secretion. Still, a number of SYL9 variants, for which we predict rather high peptide similarity to SYL9, hardly induce an IFNγ response, e.g., A7S, A7V, T5A mutants given in the upper left corner of Fig. 2. Part of this discrepancy is due to the fact that our model is not position specific, and thus underestimates the effect of mutations in the central positions, which are crucial for T cell recognition (see Fig. 1A). When more data becomes available, the peptide similarity model can be extended with a weighting accounting for the relative importance of the peptide positions.

We were able to achieve similar performances while testing the model on other peptide scanning data, e.g. La Rosa et al. [19] (HLA-A2 restricted CMV epitope, data not shown). Thus, our model was able to predict cross-reactivity of T cell clones measured in at least two different peptide-scanning library studies.

### Analysis of known cross-reactive epitopes

**Figure 3:**
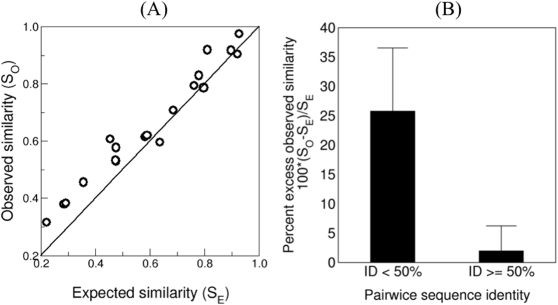
Predicted similarity of pairs of cross-reactive epitopes reported in the literature. (A) The observed similarities S_O_ between 19 cross-reactive epitopes listed in table 1 versus the expected similarities S_E_ (for definitions of S_E_ and S_O_ see the text). The line depicts the diagonal S_O_ = S_E_. 16 out of 19 points fall above the diagonal. (B) The relative increase of observed similarity with respect to the expected similarity. There is a strong inverse relationship between sequence identity and the level of conservation on non-identical positions. Cross-reactive epitopes with low sequence identity share increasingly conserved amino acids on the remaining positions.

Striking examples of T cell cross-reactivity have been reported for CTL responses to viruses [17], [20]. It was shown that CTLs that were elicited during a primary viral infection might also respond when the same mice are re-infected with unrelated viruses. By mapping the different viral epitopes to which a particular T cell clone can respond, it was demonstrated that these cross-reactive epitopes can share very little sequence identity [17], [20] leading to the conclusion that CTLs are extremely non-specific [8], [17], [20]. Reviewing the literature, we compiled a set of 19 cross-reactive epitopes in Table 1. These epitopes are restricted to the K^b^, K^d^, D^b^, HLA-A1, HLA-A2, and HLA-B62 MHC alleles. Some of the epitopes share only a few amino acids; one is even different on all positions, while others share the majority of the amino acids. We assumed that the first epitope (*x*) in a cross-reactive pair (*x,y*) is the original epitope for which the cross-reactive CTL clone was first raised, and that it was observed to respond to y later (see Table 1). To test whether these cross-reactive epitopes that differ markedly in their sequence could nevertheless have structurally similar amino acids on the non-identical positions, we did the following. First we computed the similarity of the cross-reactive epitopes *S_O_*. Then we constructed an ensemble of random peptides that have the same identical positions as the cross-reactive epitope pair but otherwise consist of random amino acids (see Materials and Methods for details). We then computed the baseline (or the expected) peptide-similarity as the average random similarity denoted *S_E_*. In 16 out of 19 pairs the observed similarity *S_O_* exceeded the expected baseline similarity *S_E_* (see Table 1 and Fig. 3A, p<0.02, Fisher's exact test). Fig. 3A shows the observed (*S_O_*) versus the baseline expected similarity (*S_E_*) and the solid line presents the case where *S_O_ = S_E_*. This plot demonstrates that cross-reactive epitopes are significantly more similar than unrelated peptides with the same level of sequence identity. Thus, in cross-reacting T cell ligands non-identical positions are significantly more conservative than random. Fig. 3B shows this more explicitly. The 19 epitope pairs were split in two groups according to the level of sequence identity; *less than 50%* and *larger than or equal to 50%* identity. For both groups we compute the *percent excess observed similarity* of the cross-reactive constituents defined as 100⋅(*S_O_*−*S_E_*)/*S_E_* From Fig. 3, we clearly see that for “seemingly” unrelated sequences (identity<50%) the excess observed similarity (y-axis) is on average 25.8% +/− 10.8%, i.e., when sequence identity is low, the observed similarity is much higher than the expected similarity. Conversely, for epitopes sharing more than half the amino acids, excess similarity drops markedly (2.0% +/− 4.3%) probably because cross-reactivity is maintained by the more numerous identical positions. The difference in excess observed similarity between the groups is highly significant (p<0.001, rank test), which suggests that amino acid identity is a poor measure for estimating physicochemical similarity, and thus T cell cross-reactivity. In summary, the above results demonstrate that biochemical similarity plays a large role in defining CTL cross-reactivity when sequence identity is low. In such cases, cross-reactivity is observed for non-identical, but conservative, substitutions preserving structural and/or physiochemical properties satisfying the idiosyncratic binding constrains of the responding TCR.

**Table 1:** Examples of cross-reactive epitopes reported in the literature.

MHC	Initial	Subsequent	Initial	Subsequent	Overlap	Id	S_O_	S_E_	Ref.
H2-Kd	LCMV NP	PV NP	**YTVKYPNL**	**YTVKFPNM**	**YTVK.PN.**	6/8	0.92	0.81	[27]
H2-Kd	LCMV NP	VV P1	**YTVKYPNL**	**YNSLYPNV**	**Y...YPN.**	4/8	0.71	0.68	[17]
H2-Kd	LCMV NP	VV P10	**YTVKYPNL**	**STLNFNNL**	**.T....NL**	3/8	0.58	0.48	[17]
H2-Kd	LCMV NP	VV A11R	**YTVKYPNL**	**AIVNYANL**	**..V.Y.NL**	4/8	0.61	0.58	[28]
H2-Kd	LCMV NP	VV A11R	**AVYNFATC**	**AIVNYANL**	**A..N.A..**	3/8	0.61	0.45	[28]
H2-Kd	LCMV NP	VV A11R	**ISHNFCNL**	**AIVNYANL**	**...N..NL**	3/8	0.53	0.48	[28]
H2-Kd	RSV5 M2-82	RSV M2-71	**SYIGSINNI**	**EYALGVVGV**	**.Y.......**	1/9	0.46	0.36	[29]
H2-Kd	CTL agonist (APL)	IGRP206-214	**KYNKANWFL**	**VYLKTNVFL**	**.Y.K.N.FL**	5/9	0.6	0.64	[30]
H2-Kd	Dengue 2 NS3-298	Dengue 3 NS3-299	**GYISTRVEM**	**GYISTRVGM**	**GYISTRV.M**	8/9	0.9	0.92	[31]
HLA-A2	EBV BMLF1-280	FLU A M1-58	**GLCTLVAML**	**GILGFVFTL**	**G....V..L**	3/9	0.53	0.47	[32]
HLA-A2	EBV BMLF1-280	FLU A NP-85	**GLCTLVAML**	**KLGEFYNQM**	**.L.......**	1/9	0.38	0.28	[20]
HLA-A2	EBV BMLF1-280	EBV LMP2	**GLCTLVAML**	**LLWTLVVLL**	**.L.TLV..L**	5/9	0.62	0.59	[20]
HLA-A2	EBV BMLF1-280	EBV BRLF1	**GLCTLVAML**	**YVLDHLIVV**	**.........**	0/9	0.32	0.22	[20]
HLA-A2	FLU A NA-231	HCV NS3-1073	**CVNGSCFTL**	**CVNGVCWTV**	**CVNG.C.T.**	6/9	0.83	0.78	[33]
HLA-A2	FLU A M1-58	EBV EBNA3A-596	**GILGFVFTL**	**SVRDRLARL**	**........L**	1/9	0.38	0.29	[20]
HLA-A2	HPV 16 E7-11	Coronavirus NS2-52	**YMLDLQPET**	**TMLDIQPED**	**.MLD.QPE.**	6/9	0.79	0.76	[34]
HLA-A2	HIV ENV GP-120	M. tuberculosis	**VPTDPNPPEV**	**VLTDGNPPEV**	**V.TD.NPPEV**	8/10	0.79	0.8	[35]
HLA-B62	Dengue 2 NS3-71	Dengue 3 NS3-71	**DVKKDLISY**	**SVKKDLISY**	**.VKKDLISY**	8/9	0.92	0.9	[36]
HLA-A1	Hantaanvirus (Sin)	Hantaanvirus (Seoul)	**ISNQEPLKL**	**ISNQEPMKL**	**ISNQEP.KL**	8/9	0.97	0.93	[37]

The columns are as follows: 1) MHC restriction, 2) source pathogen and protein for initial infection, 3) source pathogen and protein for subsequent infection, 4) original epitope of initial infection, 5) cross-reactive epitope for subsequent infection, 6) sequence overlap between the cross-reactive epitopes, 7) sequence identity (Id), 8) observed peptide similarity *S_O_* and 9) expected peptide similarity *S_E_* (for definitions of *S_O_* and *S_E_* see the main text) 10) reference to the experimental work. Some infectious agents are indicated with abbreviated names and these are: LCMV: Lymphocytic choriomeningitis virus, PV: Pichinde virus (PV), VV: Vacinia virus, EBV: Epstein-Barr virus, RSV: Respiratory synthical virus, HCV: Hepatitic C virus, and HPV: Human papiloma virus.

### Non-immunogenic HIV peptides tend to be more similar to human self-antigens

**Figure 4:**
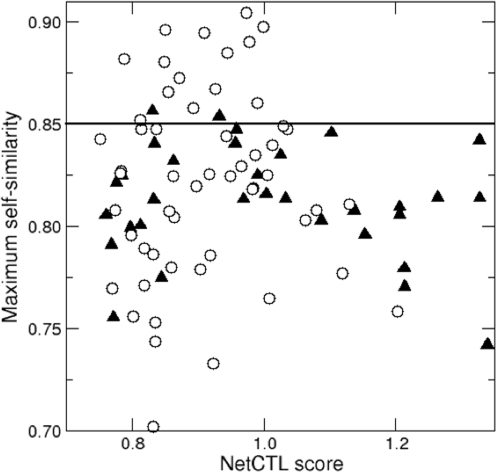
HIV self-similarity and immunogenicity. The NetCTL antigen presentation score (x-axis) and maximal self-similarity (y-axis). Confirmed HIV epitopes are shown as black diamonds and predicted, non-immunogenic HIV peptides are shown as open circles. The 0.85 self-similarity line described in the text divides the y-axis. The region above the line is clearly dominated by non-immunogenic antigens where 88% (14/16) are non-immunogenic compared to the expected frequency of 62% (54/87). This difference is significant (p<0.05, Fisher's exact test). Peptide similarity was calculated using the BLOSUM35 matrix.

Another open question in T cell response is why roughly half of all foreign cell surface-presented antigens fail to raise a T cell response [3], [4], [21]. Tolerance to self-antigens could explain this lack of immunogenicity, in which case the degree of similarity to self-antigens should predict which foreign antigens are likely to be non-immunogenic. We examined this effect of self-tolerance on immunogenecity using our cross-reactivity model. First, a large set of self-antigens was defined, and secondly, a list of non-self (e.g., HIV) antigens was built, labeled as either immunogenic or non-immunogenic according to experimental evidence (data obtained from the Los Alamos HIV database, see Materials and Methods). The expectation was that T cell clones, with high affinity for HIV peptides similar to self peptide(s), have been tolerized during thymic education via negative selection [22], [23]. Such TCRs should therefore not be present in the functional T cell repertoire thus causing tolerance to molecular mimics of self-peptides. We define a score of cross-reactivity to self as the maximum peptide similarity between the non-self antigen and the set of all self-antigens (see Materials and Methods) and test whether non-immunogenic peptides have a higher cross-reactivity score to self when compared to immunogenic ones. We downloaded the human proteome from the NCBI website and identified a set of 230,460 potential HLA-A2 self-antigens (see Materials and Methods). Next, we downloaded the HIV proteome from the Los Alamos HIV database and predicted a set of potential HLA-A2 epitopes. 33 of the 91 predicted HIV candidate epitopes were annotated as A2 supertype restricted epitopes in the Los Alamos database of CTL HIV epitopes, while the remaining 54 of the HIV peptides were never identified as epitopes. Another four peptides were found to be immunogenic for other HLA alleles than HLA-A2. Since it would be wrong to tag these epitopes as “non-immunogenic”, they were excluded from the data set. The 33 confirmed HLA-A2 epitopes were labeled: *confirmed HIV epitopes* and the remaining 54 possible non-immunogenic peptides were given the label: *putative, non-immunogenic HIV peptides*. It is possible that future studies reveal that a number of the putative non-immunogenic HIV peptides do in fact elicit CTL responses in HLA-A2^+^ patients. Nevertheless, this set of HIV peptides should be enriched in HIV peptides that fail to generate CTL responses. Maximal similarity scores were computed between all 87 HIV peptides (33 immunogens and 54 putative non-immunogens) and the set of 230,460 predicted HLA-A2 self-epitopes. Fig. 4 shows a scatter plot of the 33 HIV immunogens (black diamonds) and 54 putative non-immunogens (open circles). The x-axis shows the predicted antigen presentation score (NetCTL) while the y-axis shows the estimated maximum similarity to the self-antigens *S_SELF_(x,y)* (see Materials and Methods). Immunogenic peptides tend to be less self-like, although the difference between immunogens and non-immunogens is not significant (p = 0.2, Mann-Whitney). Drawing a horizontal line at y = 0.85 separates the most self-similar HIV antigens from the rest (the results presented in Fig. 2B suggest that IFNγ response would fall to a half when the similarity drops to 85%.) For the antigens that have a self-similarity score above 0.85, most (14/16) are classified as non-immunogenic HIV antigens i.e. predicted epitopes not confirmed by experimental evidence (p-value<0.05, Fisher's exact test). Note, that the NetCTL score does not correlate with the maximal self-similarity score (p-value = 0.42, exact estimate) and the above difference between the immunogenic and non-immunogenic antigens is therefore not explained by the difference in the NetCTL scores. Repeating our analysis for HLA-A3 and HLA-B7, we found similar tendency of more-self-likeness among non-immunogenic HIV-1 peptides (p<0.3 and p<0.45 respectively). Summarizing, these results suggest that similarity to self-antigens plays a role in discriminating immunodominant from cryptic peptides.

## Discussion

Many studies have suggested that T cells can recognize totally unrelated peptides and a new term, **poly-specificity** was coined to express the high specificity of T cell receptors to unrelated peptides [6]. The “unrelatedness” of the peptides was defined as low sequence identity, however, sequence identity might not be able to account for the total amount of structural similarity that drives TCR recognition. Here, we demonstrate that this is indeed the case: T cell receptors recognize biochemically and structurally related peptides and cross-reactivity is, up to a degree, predictable. The loss of recognition simply depends on the number and similarity of non-identical amino acid between cross-reactive constituents. We find that the majority of the seemingly “unrelated” cross-reactive peptides have a significantly higher biochemical similarity to each other than what would be expected from truly “unrelated” peptides. This is especially true for peptides with very limited identity. To our knowledge this is the first study that analyzes a large set of cross-reactive peptide-MHC combinations and demonstrates that the cross-reactivity can, up to a certain extent, be predicted.

Because negative selection of immature thymocytes remove high affinity TCR specific for self-antigens [7], we expected that this should leave a “hole” in the T cell repertoire around negative selecting self-antigens. Hence, if an infected cell presents a nonself antigen that is highly similar to a negative selecting self-antigen, then this foreign antigen might not be matched by any available TCR which could provide an explanation for why around half of foreign pMHC do not generate a T cell response [3], [4], [21]. We tested this hypothesis for HLA-A2 restricted HIV-1 response and showed that the absence of T cell response to part of the non-confirmed (i.e. putative non-immunogenic) HLA-A2 restricted HIV-1 peptides can be explained by their similarity to self antigens. These results are in agreement with a recent study by Rolland et al. [24], who showed a trend of more-self-likeness (measured in terms of number of shared amino acids) among HIV peptides with no detectable CTL responses in a large study group. We predict that the correlation found by Rolland et al. would be stronger if amino acid similarity is taken into account.

If peptide similarity can be used to describe T cell reactivity, what would then be the best model to describe similar peptides? We have chosen for the simplest model (given by Eq. (2) in Materials and Methods) because systemic data on cross-reactive peptides is very limited. An obvious extension of this model would be to add position dependence, i.e., account for the fact that central positions play a larger role.

The number of potential antigens exceeds the number of T cells in the immune system and the ability to recognize multiple ligands is required to mount at least a few responses to all potential pathogens [8]. Here, we demonstrate that the number of expected T cell ligands is not necessarily reduced by restricting T cell recognition to cover only similar peptides: it is still possible for a T cell to recognize 10^5^−10^6^ peptides. These estimates of the complexity of CTL recognition are well within the bounds of earlier estimates [8]. In summary, the results presented here quantify, to our knowledge for the first time, that the basis of T cell recognition is amino acid similarity, defined in terms of biochemical properties of amino acid side chains.

## Materials and Methods

### TCR binding motif

Lee et al. [10] analyzed the specificity of CTL responses against the immunodominant HLA-A2 restricted HIV Gag epitope SLFNTVATL (SFL9). IFNγ production was measured in response against all 171 single mutant variants of SFL9 for two T cell clones (G10 and T4), and for purified Peripheral Blood Mononuclear Cells (PMBCs) using an ELISPOT assay. Purified PBMCs consisted of just two clones where one was dominant. CTL responses were reported as the percentage of maximal IFNγ (I) obtained for the reference ELISPOT of SFL9 and discretized in five intervals: ]0;30], ]31;70], ]70;100[and 100%. We replaced each interval by the interval midpoint, translating the original data to the real values: 0.00, 0.15, 0.50, 0.85 and 1.00 respectively. We defined a measure of the “relative frequency of recognition”, *f_i,a_*, for a variant carrying mutation *a* on position *i*, as the response of the variant, divided by the total of all variants on the same position as:
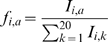



### Simpson index

To measure the diversity at position *i* in an alignment, we define *D(i) = 1/S(i),* where *S*(*i*) = Σ^20^ *_a_*_ = 1_(*p_i,a_*)^2^ is the Simpson index. Here, *p_i,a_* is the probability that a particular amino acids occurs at position *i* in the alignment where Σ^20^ *_a_*_ = 1_ *p_i,a_* = 1. In all cases *p_i,a_* = *f_i,a_* (see above definition). If position *i* is fully conserved, then *D(i) = 1*, if all amino acids occur with equal frequency i.e. *p_i,a_* = 1/20, then *D(i) = 20*.

### Peptide similarity score

The un-normalized peptide similarity score *A(x,y)* between reference epitope: *x* = {*x*_1_, L, *x*_N_} and peptide: *y* = {*y*_1_, L, *y*_N_} of the same length *N* is defined as the sum of substitution scores along the sequences expressed by the relation:
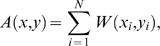
(1)
*_W_*_(*xi*, *yi*)_ is the amino acid substitution matrix, e.g., BLOSUM35, providing a measure of how conservative substitutions are. The peptide-similarity score for the reference peptide *x* spans the interval: *I^x^* = [*A^x^*_min_; *A^x^*_max_] where the length of the interval *|I| = A*_max_−*A*_min_ depends on references peptide *x* and matrix *W*. Two different intervals (*I^x^, I^y^* ^≠*x*^) are not comparable *per se*. Thus, we define the normalized peptide similarity using the relation
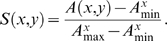
(2)


This equation constitutes the model of peptide similarity used throughout subsequent analysis. *S(x,y)* measures how much peptide *y* resembles *x* in terms of the number and magnitude of conservative substitutions. *A^x^*_max_ is the auto-peptide-similarity score of *x*. Thus, *A^x^*_max_ = *A(x,x)*. If peptide *y* is a mimic of *x* then *S(x,y)* should be close to 1. The other extreme value (*A^x^*_min_) is found by comparing *x* with a peptide *x̅*, where on each position the amino acid in *x̅* corresponds to the substitution in *x* with the smallest value i.e. the least likely substitution. In this way 0≤*S*(*x*,*y*)≤1 for all peptides *y*. The peptide-similarity score is asymmetric i.e. *S*(*a*,*b*)≠*S*(*b*,*a*) despite *W* being symmetric. The reason is that the extreme values (*A*_min_,*A*_max_) cannot be guarantied to be identical for any pair of peptides (*a,b*). Reference peptides *x* which are dominated by amino acids like tryptophan that hardly ever substitute, will have few highly similar peptides (*y*) which satisfy the condition: *S(x,y) ≈ 1*. In contrast, reference peptides which are enriched in amino acids that are more likely to substitute (given the matrix *W*) have a greater number of highly similar peptides. This property is captured by the asymmetry of *S.*

### Expected baseline similarity between primary epitopes and presumably unrelated peptides

The observed similarity *S_O_* of pairs of experimentally verified cross-reactive epitopes (*x,y*) is to be compared to unrelated peptides *(z_i_*) which retain the sequence identity of (x,y) but have an otherwise random amino acid on non-identical positions. The procedure to compute the “unrelated” or “baseline” expected similarity is best illustrated by an example: The HLA-A2 epitopes *x* = GLCTLVAML and *y* = GILGFVFTL from EBV and influenza-A share 3 identical positions: G1, V6 and L9. We first compute the observed similarity *S_O_ = S(x,y)* between epitopes *x* and *y* using Eq. (2). Then we generate a set of *N = 10.000* random peptides, *z* ^1^, *z* ^2^, L, *z^N^*, with the same identical positions, i.e. we have *z_i_* = G……V…L where a dot can be any amino acids avoiding identity with *x* at that position. The expected similarity between the primary (original) epitope (*x*) and the unrelated but semi-identical artificial peptides *z* is then defined as the average similarity to the ensemble of unrelated peptides as: 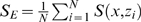.

### Non-immunogenic HIV-peptides (HLA-A2)

The HIV-1 HXB2 sequence for Env, Pol, Vpu, Rev, Tat, Vif, Vpr, p17, p24 and p2p7p1p6 and the HIV-1 clade B consensus sequence for Nef (due to a stop codon in HXB2-Nef) were downloaded from the Los Alamos database (www.hiv.lanl.gov). There was also one stop codon in HXB2 sequence for TAT, however, no TAT peptides were predicted to be HLA-A2 epitopes and thus the stop codon did not interfere with our results. Out of 3,063 HIV nonamers, 91 were predicted to be HLA-A2 epitopes using NetCTL version 1.2 [25], [26] and default selection threshold (0.75). NetCTL predicts the level of antigen presentation by combining three separate predictions of: proteasomal cleavage, TAP affinity and MHC biding. Four out of the 91 predicted HIV epitopes were found to be immunogenic for other supertypes than HLA-A2 and were filtered out. These were: QLQARILAV, RILAVERYL (class II, DPW4.2), TLYCVHQRI (HLA-A11) and SINNETPGI (HLA-A25). Of the remaining 91-4 = 87 peptides, 33 were confirmed HLA-A2 epitopes by cross-referencing the records of the LANL CTL epitope summary table (downloaded December 2006). Thus, the epitope prediction resulted in the identification of 87 possible HLA-A2 restricted HIV epitopes where 33 (38%) were confirmed and 54 (62%) were not.

### Human HLA-A2 restricted self-antigens

The human proteome was downloaded from the NCBI website (www.ncbi.nlm.nih.gov/Genomes/ date: 29 march 2006) and contained 34.460 protein sequences. The removal of proteins containing the words: *predicted*, *hypothetic* or *isoform* in the protein description label lead to a final core human proteome of 14,034 human protein sequences. We predicted A2 self-antigens using NetCTL version 1.2 [25], [26] for all these protein sequences (default epitope selection threshold). Repeats were removed, along with a small set of self-peptides, which contained the unknown amino acid (X). The final set consisted of 230,460 predicted human HLA-A2 restricted self-antigens each of length 9.

### HIV self-similarity (HLA-A2)

The maximal similarity between predicted HIV antigens (*x*) and the set of human 230,460 self-antigens (*y*) was defined as the self-similarity score *S_self_*(*x*) = max(*S*(*x*,*y*)) for HIV peptide *x*. Self-similarity scores were obtained for all confirmed HIV epitopes and putative HIV antigens. Because no identical matches were found between HIV epitopes and self-antigens, self-similarity scores were always *S(x,y)<1*. Confirmed HIV epitopes and putative non-immunogenic HIV peptides were ranked on maximum self-similarity, and the combined ranking was split in two parts: a) The peptides with a self-similarity score greater than 0.85 and b) and peptide with a self-similarity score below 0.85. We used Fisher's exact test to compute the significance of the difference in the frequency of putative HIV epitopes in the top versus the bottom.
